# Neuro-Muscular Differentiation of Adult Porcine Skin Derived Stem Cell-Like Cells

**DOI:** 10.1371/journal.pone.0008968

**Published:** 2010-01-29

**Authors:** Dominik Lermen, Erwin Gorjup, Paul W. Dyce, Hagen von Briesen, Paul Müller

**Affiliations:** 1 Department of Biogeography, Trier University, Trier, Germany; 2 Department of Cell Biology and Applied Virology, Fraunhofer Institute for Biomedical Engineering, St. Ingbert, Germany; 3 Department of Animal and Poultry Science, University of Guelph, Guelph, Ontario, Canada; Health Canada, Canada

## Abstract

**Background:**

Due to the genetic relationship to humans, porcine stem cells are a very important model system to investigate cell differentiation, associated cell signaling pathways, and cell fate. Porcine skin derived stem cells have been isolated from mid-gestation porcine fetus recently. To our knowledge, stem cells from the skin of the adult porcine organism have not been isolated until now. Hence, to our knowledge, we here describe the isolation, expansion, characterization and differentiation of multipotent porcine skin derived stem cell-like cells (pSSCs) from the adult porcine organism for the first time.

**Methodology/Principal Findings:**

pSSCs had a spindle shaped morphology similar to mesenchymal stem cells (MSCs). They could be maintained proliferatively active in vitro for more than 120 days and were able to form colonies from single cells. pSSCs expressed Sox2 and Oct3/4, both transcription factors essential to the pluripotent and self-renewing phenotypes of embryonic stem cells, which recently gained attention due to their function in inducing pluripotent stem cells. Furthermore, the expression of the progenitor marker nestin, the somatic stem cell markers Bcrp1/ABCG2, Bmi1, and Stat3 was detected by reverse transcriptase-polymerase chain reaction (RT-PCR) in undifferentiated pSSCs. Flow cytometry revealed the expression of the MSC related proteins CD9, CD29, CD44 and CD105, but not CD90. After neuronal differentiation cells with a characteristic morphology of neuronal and smooth muscle-like cells were present in the cultures. Subsequent immunochemistry and flow cytometry revealed the down-regulation of nestin and the up-regulation of the neuron specific protein beta-III-tubulin and the astrocyte marker GFAP. Also, alpha-SMA expressing cells increased during differentiation suggesting the neuro-muscular differentiation of these skin derived cells. pSSCs could also be induced to differentiate into adipocyte-like cells when cultured under specific conditions.

**Conclusions/Significance:**

Adult porcine skin harbors a population of stem cell-like cells (pSSCs) that can be isolated via enzymatic digestion. These pSSCs show characteristic features of MSCs originated in other tissues and express the embryonic stem cell marker Oct3/4, Sox2, and Stat3. Furthermore, pSSCs have the potential to differentiate into cells from two different germ lines, the ectoderm (neurons, astrocytes) and the mesoderm (smooth muscle cells, adipocytes).

## Introduction

Since it is widely known, that pig skin has similar histological and physiological properties as human skin, it is becoming increasingly important as an in vitro model. The easy accessibility of porcine skin and its broad spectrum of applications as a tissue source for scientific experiments permits a wide range of biological research questions to be studied, with regard to physiological, anatomical, toxicological, and developmental properties [1]–[3]. Furthermore, porcine skin provides an easy accessible source of tissue for the isolation of cells including adult stem cells. Small skin biopsies are sufficient and can be obtained in a minimal invasive way. Besides embryonic stem (ES) cells from pigs [4]–[6], adult stem cells [7]–[9] from large animals such as the pig offer a great potential to investigate cell differentiation, cell fate, and the associated cell signaling pathways involved in cell differentiation.

Skin harbors a variety of stem cells in the epidermis [10]–[16], dermis [17]–[22], including appendages such as sebaceous glands [23] and the hair follicle [24], [25]. All these types of skin-derived adult stem cells are interesting candidates for human therapeutic applications. Also, conservation biologists recognize a huge potential in adult stem cells as candidate cells for conservation measures of endangered animals [26]–[29].

Regarding porcine skin derived stem cells until now only cells from the fetal organism were studied. Multipotent porcine stem cells, that can differentiate into neuronal and adipose cells, have been isolated from fetal porcine skin [30]. These cells, termed PSOS (porcine skin originated sphere) cells, proliferate as spherical aggregates of cells and grow under serum free conditions. These cells are also able to form oocyte-like cells when cultured in the presence of specific medium containing follicular fluid [31].

Stem cells share a set of characteristics that indicate pluripotency or other stem cell related features like unrestricted proliferation and maintenance of an undifferentiated state. For instance, the pluripotency of embryonic stem (ES) cells is mainly maintained via a transcriptional network of factors that regulate a multiple set of transcription factors. The most essential transcription factors that are involved in this regulatory complex of embryonic stem cells are Oct3/4 and Sox2 which can act synergistically. Their synergistic expression and activation enhances an Oct-Sox-complex that leads to the transcription of target genes like Nanog, Kfl4, Lefty1 or Fgf4 which are know to be involved in many development related processes and in the maintenance of stem cell associated properties [32]–[36]. Regarding murine ES cells the transcription factor Stat3 plays a major role in maintaining pluipotency. It is known to be activated through epidermal growth factor (EGF) [37] or leukemia inhibitory factor (LIF)[38], [39]. LIF binds to gp130 and activates a gp130-Jak-Stat signaling pathway that finally leads to the phosphorylation of the Stat3 protein that subsequently migrates into the nucleus and regulates important gene transcription processes [40], [41].

The expression of Oct3/4, Sox2 and Stat3 is also described for adult stem cells what underlines their importance not just for the maintenance of stem cell properties in embryonic stem cells but also in stem cells from adult organisms [17], [42], [43]. Besides the hematopoietic stem cells, bone marrow mesenchymal stem cells (BM-MSCs) are the best investigated adult stem cells. These adherent growing cells have a spindle shaped fibroblast-like morphology and are able to form cell-colonies from single cells. Furthermore, they are known to express a huge set of cell surface proteins, for example CD9 (Tetraspanin), CD29 (β1-Integrin), CD44 (Hyaluron), CD71 (Transferrin receptor), CD73 (SH3/4), CD90 (Thy-1), CD105 (Endoglin), CD106 (VCAM-1) and more [44]–[46]. Interestingly, none of those surface proteins identify the “stemness” of BM-MSCs, but many of them are generally investigated and utilized for a phenotypic characterization of adult stem cells [17], [43], [45]. In addition, intracellular components have been identified as being typical for adult stem cells of different tissue of origin, not just of BM-MSCs. The intracellular filament protein Nestin is widely discussed as an adult stem cell or progenitor marker [18], [47]–[49]. Bmi1, a polycomb group repressor, was first identified as an important component of the self-renewing mechanism of murine hematopoietic stem cells. It is now known to be expressed in a huge variety of adult stem cells. Moreover, Bmi1 is evolutionary highly conserved and can be identified in stem cells of different animals. It contributes to the maintenance of stem cell related properties via the repression of p16^Ink4a^, p19^Arf^ and p53, all related to the process of cellular senescence [50]–[54]. The ABC transporter ABCG2 or its murine ontholog Bcrp1 may be regarded as another somatic stem cell marker. Adult stem cells of different tissues of origin can be isolated via an incubation with the dye 33342 (Hoechst) and a subsequent fluorescent cell sorting with regard to cells that efflux the dye and appear as side population [7], [55], [56]. These side population cells express ABCG2/Bcrp1[57]. This transporter operates as dye pump and therefore is responsible for the efflux of the incorporated dye 33342 [58].

BM-MSCs have the potential to differentiate along the adipogenic, osteogenic and chondrogenic lineage [44], [45], [59]–[64]. Latest investigations also describe their differentiation into neuronal cell types as well as muscle-like cells and revealed the expression of proteins related to differentiated cells like GFAP and β-III-tubulin (neurons, astrocytes) and α-SMA (smooth muscle cells). This describes their tansdifferentiation potential into cells from different gem layers [59], [65]–[69].

To our knowledge, in this report we describe the isolation, expansion, phenotypic characterization and differentiation of stem cell-like cells derived from adult porcine skin for the first time. The possibility to isolate stem cells from adult instead of fetal porcine skin provides a much easier accessible *in vitro* model system to investigate the properties and opportunities of non-embryonic stem cells for possible future therapeutic applications.

## Results

### Cell Isolation and Cultivation

**Figure 1:**
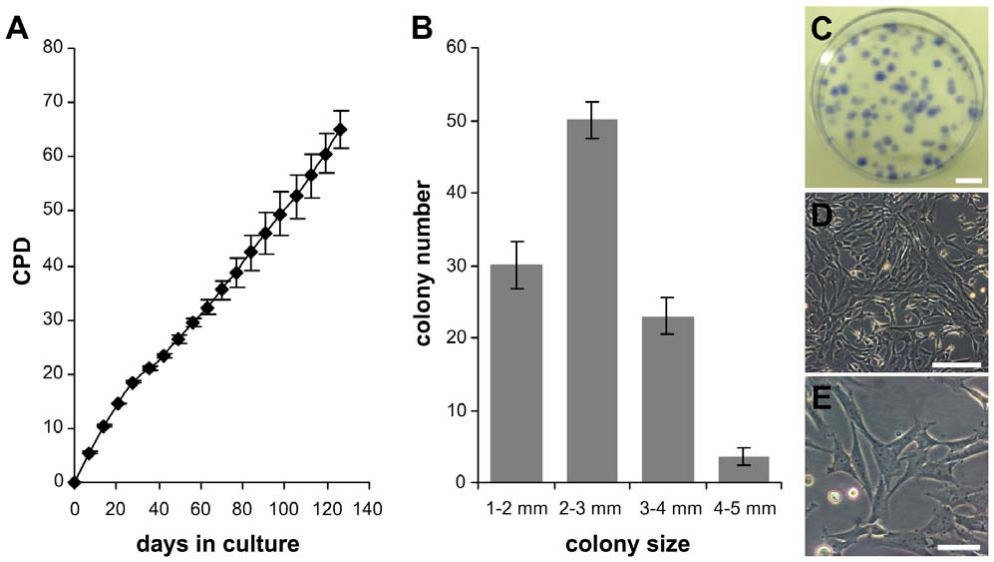
Growth characteristics of pSSCs in vitro. (A) Proliferation of pSSCs: Given is the CPD (cumulative population doubling) of pSSCs as function of days in culture. Data are expressed as means ± SEM from three independent pSSC strains. (B) Average CFU efficiency of one representative pSSCs strain after 22 CPD. Mean of three experiments ± SEM. (C) Image of cell colonies (stained with trypan-blue) formed after 11 days seeded with 10 cells per cm^2^ growth surface. Scale bar: 1 cm. (D, E) Morphological appearance of pSSCs. Scale bars: (D) 200 µm, (E) 100 µm.

Single cells were isolated via enzyme digestion. The cells were plated initially at a high density with 2×10^5^ cells per cm^2^ growth surface in stem cell medium (SCM). After 1–4 days cells adhered to the plastic surface of the flask and started to proliferate. Due to the whole skin biopsy including the epidermis and dermis being digested, many different cell types including keratinocytes adhered. The incubation of the isolated cells with 0.25% trypsin/EDTA at 37°C for up to 5 min revealed a temporal displaced detachment of different cell types. Stem cells detached from the surface after an incubation time of ∼60–90 sec while the undesired keratinocytes and differentiated cells remained adhered at this point in time. This process was used to purify the pSSCs. After reseeding of the target cells, this purification step was repeated 2–4 times until a morphologically consistent population of the purified cells was verifiable by microscopic inspection. pSSCs were then routinely seeded at 3,000 cells per cm^2^ in tissue culture flasks. They had a mean diameter of 14.57 µm (in suspension) and appeared morphologically as small, spindle shaped, fibroblast-like cells (Fig. 1D, E).

### Proliferation

To examine the proliferation capacity, pSSCs were seeded in SCM and passaged every seven days, when the cells reached ∼90% confluence. We observed that the pSSCs cultured under the described conditions can be passaged at least 18 times during 126 days of in vitro culture. During the culturing there was no detectable significant change in either morphology or proliferation rate. Following 126 days of culture the experiment was ceased while the pSSCs of all three animals underwent 64,9 (±3,4 SEM, average of three independent pSSC strains) population doublings and were still proliferatively active (Fig. 1A). Hence, pSSCs have a population doubling time of ∼47 hours. Cryopreservation did not change their growth pattern and morphology (data not shown).

### CFU Efficiency

Since one important indicator for the presence of stem cells is the ability of cells to easily form colonies [70], the colony forming unit efficiency (CFU) of the isolated pSSCs was investigated. The results showed clearly that pSSCs are able to form colonies (Fig. 1C). The colony forming unit efficiency of one representative pSSC strain at passage 5 (CPD 22) is shown in Fig. 1B. After 11 days 37.69% (±1.43) of the seeded cells were capable of forming colonies larger than 1 mm in diameter. Of these colonies, 30% (±3.21) had a diameter of 1–2 mm, 50% (±2.52) a diameter of 2–3 mm, 23% (±2.52) a diameter of 3–4 mm, and 3.67% (±1,20) had a diameter larger than 4 mm.

### Phenotypic Characterization of pSSCs

To examine the expression of stem cell related marker genes of pSSCs that indicate proliferation capacity and the inhibition of differentiation, RT-PCR analysis was performed regarding the expression of nestin, Bcrp1/ABCG2, Bmi1, Stat3, Sox2 and Oct3/4.

**Figure 2:**
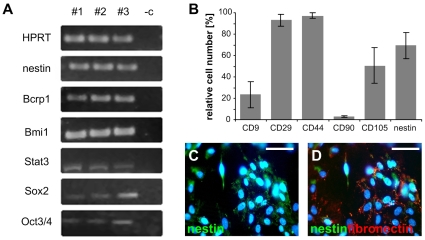
Expression of stem cell related genes and translated proteins. (A) RT-PCR analysis of stem cell related genes. Line 1–3: results of three independent cell strains, line 4: negative control (-RT) from one representative cell strain. (B) Flow cytometric analysis of cell surface markers CD9, CD29, CD44, CD90, CD105, and nestin. Data are expressed as means ± SEM from three independent pSSCs strains after 22 CPD. (C) Immunocytochemistry revealed a subpopulation of pSSCs expressing nestin. (D) Double staining indicates the coexpression of nestin and fibronectin. Scale bars: 50 µm.

The result of the RT-PCR analysis revealed that all the investigated genes are expressed in undifferentiated pSSCs (Fig. 2A).

Immunocytochemistry also indicated the expression of nestin and demonstrated the co-expression of nestin and fibronectin in pSSCs (Fig. 2C, D). Fibronectin is also expressed in bone marrow mesenchymal stem cells and the described co-expression of nestin and fibronectin was also found in fetal skin derived precursors and stem cells [16], [30].

Nestin expression was additionally quantified by flow cytometry whereby 69.39% (±12.25) of the isolated cells stained positive (Fig. 2B).

The flow cytometer analysis was used to examine the expression of cell surface proteins by the pSSCs. Due to most of the desired antibodies not cross-reacting with porcine cells, we investigated the expression of CD9, CD29, CD44, CD90, and CD105.

The majority of the pSSCs expressed on average high levels of CD29 (93.06%), CD44 (97.25%), and CD105 (50.59%) whereas 23.56% of pSSCs stained positive for CD9. CD90 was expressed at a very low level of 3.29% (Fig. 2B).

### Differentiation Potential

**Figure 3:**
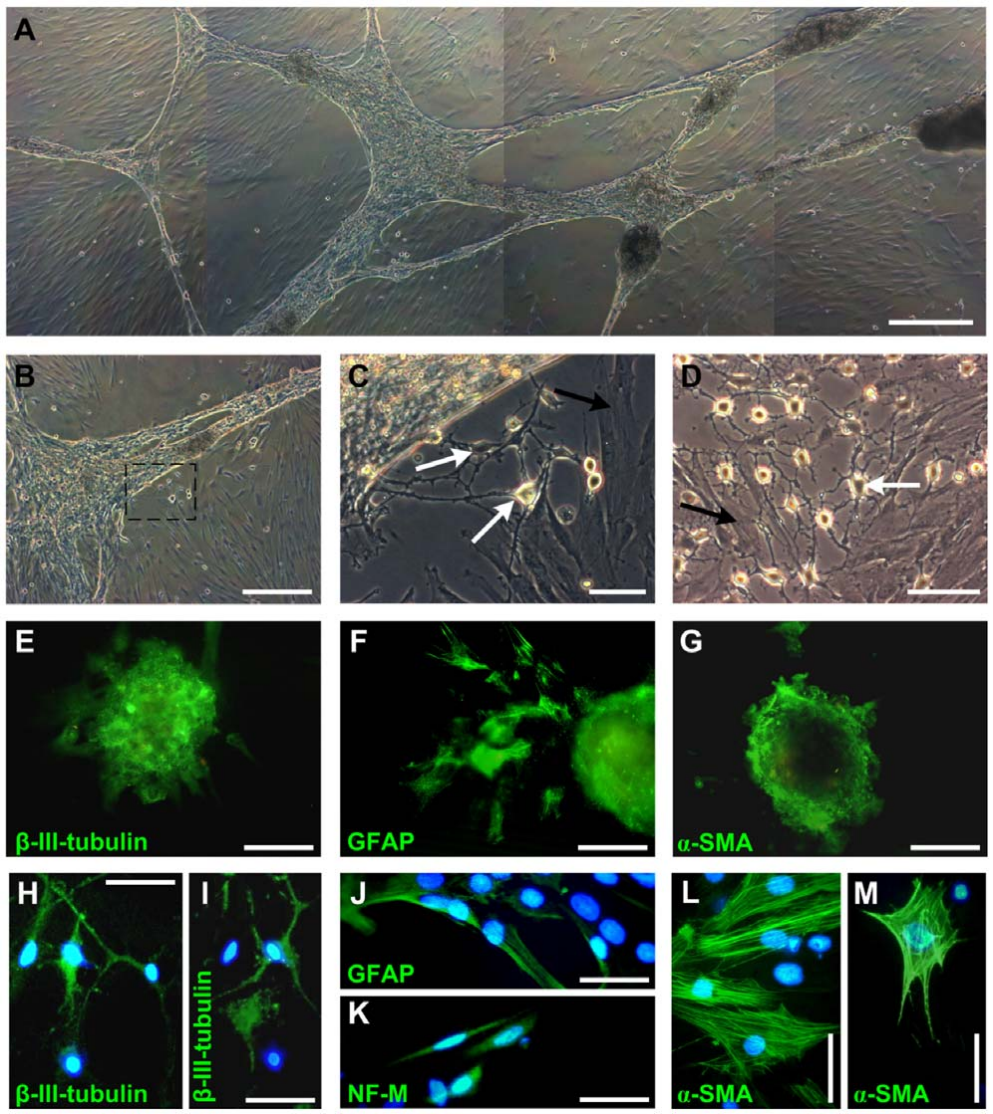
Neuro-muscular differentiation potential of pSSCs. (A) After 30 days of induced neuronal differentiation one cell strain developed a structured cell network of linked bodies. (B–D) Cells show morphologies similar of neuronal cells (white arrows) and smooth muscle cells (black arrows). Scale bars: (A, B) 500 µm, (C) 50 µm, (D) 100 µm. (C) Represents the marked region in Fig. 3B. (E–G) Immunocytochemistry revealed the expression β-III-tubulin and GFAP in the centre and some surrounding cells of the bodies, whereas α-SMA is exclusively expressed in the outer regions of the bodies and some surrounding cells. Scale bars: 50 µm. (H, I) β-III-tubulin is expressed in a subpopulation of cells that are morphological consistent with neurons. (J, K) Other subpopulations of differentiated pSSCs expressed the neuronal markers GFAP and neurofilament M. (L, M) Subpopulations with a morphology consistent of smooth muscle cells expressed the smooth muscle marker α-SMA. Scale bars: 50 µm.

Since it is known that fetal pig skin-derived stem cells can form neurons and astrocytes [16], [30], here we investigated the neuronal differentiation of pSSCs under the same conditions. Therefore, early passage pSSCs were cultured in neuronal differentiation medium (NDM). During two weeks of induced differentiation the cells' morphology changed significantly. Some cells agglomerated and formed three dimensional bodies that appeared similar to adhered neurospheres. These bodies stained positive for the neuron specific marker β-III-tubulin and GFAP, a marker for astrocytes (Fig. 3E, F). Following an additional two weeks of differentiation the cells adjacent to the formed bodies displayed the characteristic morphologies of neurons and astrocytes and expressed the neuronal lineage markers β-III-tubulin, GFAP, and the medium neurofilament (NF-M) (Fig. 3H–K).

In one pSSCs strain the three dimensional bodies connected to each other and formed a structural cell network (Fig. 3A). However, surprisingly many of the differentiating cells under these conditions flattened and evolved into a shape similar to smooth muscle cells (Fig. 3C, D; black arrows). Immunocytochemical staining of these cells revealed the expression of alpha smooth muscle actin (α-SMA, Fig. 3L, M), whereas the formed bodies showed only a marginal expression of the α-SMA (Fig. 3G).

Since cell types of two different germ lineages were present within the cultures after the neuronal differentiation, the expression of neuronal and smooth muscle lineage marker proteins and of the progenitor marker nestin were quantified. pSSCs derived from three different animals were cultured in neuronal differentiation medium for 30 days and subsequently analyzed via flow cytometry regarding their expression of nestin, β-III-tubulin, GFAP, and α-SMA. Undifferentiated cells of the same animals at the same passage were used as a reference.

**Figure 4:**
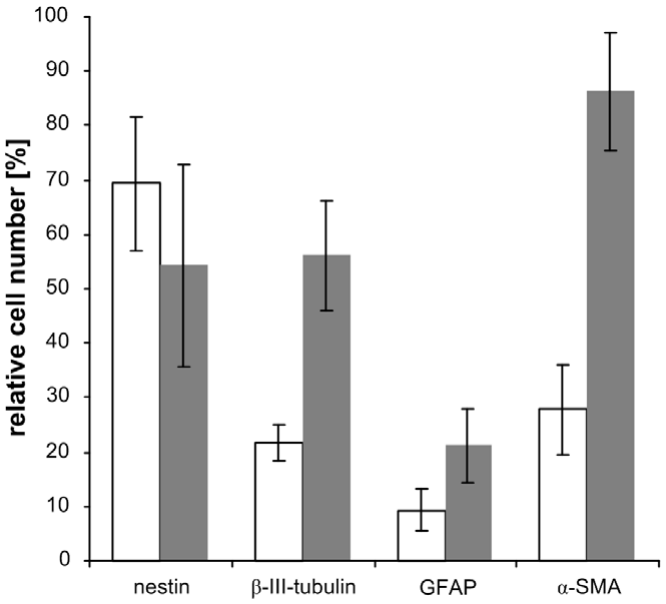
Differentiation-related regulation of proteins. Expression of the neuronal and muscular lineage markers nestin, beta-III-tubulin, GFAP, and α-SMA before differentiation (white bars) and after differentiation (grey bars) revealed by flow cytometry. Data are expressed as means ± SEM from three independent cell strains.

The resulting data revealed that besides the expression of nestin (69.39%±12.25), subpopulations of the cells already express β-III-tubulin (21.8%±3.3), GFAP (9.33%±3.72), and α-SMA (29.72%±8.26) before the initiation of the differentiation. After neuronal differentiation, nestin expressing cells decreased by an average of ∼15%, whereas β-III-tubulin expressing cells increased by an average of 21.8% to 56.14%. Also GFAP expressing cells increased by 9.33% to 21.16%. Surprisingly also α-SMA expressing cells increased on average of 29.72% to 89.35% (Fig. 4).

**Figure 5:**
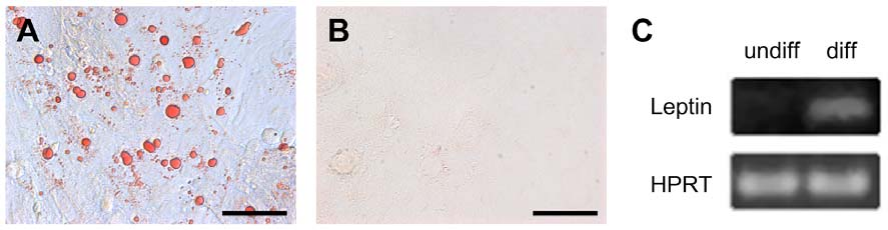
Induced adipogenic differentiation of pSSCs. (A) The adipogenic lineage differentiation is shown by bright field pictures of positive Oil Red O stained lipid vacuoles within the cytoplasm of the pSSCs after 30 days of induced differentiation. (B) Oil red O staining of pSSCs cultured in control medium. Scale bars: 50 µm. (C) Leptin expression shown by RT-PCR in adipogenic differentiated and undifferentiated pSSC cultures. Hypoxanthin-guanin phosphoribosyltransferase (HPRT) was used as an internal control for each PCR.

To analyze the differentiation potential of pSSCs to form further cell types of the mesenchymal lineage, adipogenic differentiation was induced. pSSCs were cultured in a medium according to Pittenger *et al.* [45]. The three investigated cell strains behaved very differently. After initiation of adipogenic differentiation, small lipid droplets occured in one cell strain in the following 8 days, whereas lipid vacuoles formed between days 18 and 22 in the two remaining pSSC strains. Oil red O staining demonstrated the typical phenotype of cells differentiating along the adipogenic lineage, displaying the characteristic lipid droplets (Fig. 5A). The following 30 days of adipogenic differentiation RT-PCR revealed the expression of leptin, a proteohormone released mainly by adipocytes (Fig. 5C). As a control, pSSCs were cultured in medium containing 10% FCS. No Oil Red O incorporation was detectable (Fig. 5B).

## Discussion

One major problem regarding the isolation of skin derived stem cells, particularly in the pig, is the lack of appropriate stem cell specific markers. Hence, pSSCs were isolated via enzymatic digestion and were subsequently purified via repeated short time trypsin/EDTA treatments. The isolated cells were cultured in a modified medium according to Toma *et al.* and Shih *et al.* [16], [71] which contains bFGF, EGF, and B27. Therewith, our primary cell culture conditions were selective for neurogenic differentiation of the isolated pSSCs.

To some extend, these isolated stem cell-like cells from adult porcine skin share features of mesenchymal stem cells (MSCs). The isolated pSSCs grow adherently and have a spindle-shaped fibroblast-like morphology [8], [60]. Our investigations regarding the proliferation potential and the CFU-efficiency revealed a high expansive potential of the pSSCs. Similar results were shown for human bone marrow MSCs and MSCs derived from the hair follicle [25], [70]. This makes them an interesting candidate for any genetic manipulation that could lead to a potential therapeutic or bioengineering utility. Furthermore, pSSCs express the MSC surface markers CD9, CD29, CD44, and CD105 but vary from human bone marrow MSCs in their lack of CD90 expression. Since mouse mesenchymal stem cells also lack CD90, and until now no other study investigated a CD90 expression on pig stem cells, this seems to be a species specific pattern [72], [73]. The investigated cell surface proteins are found to be expressed at different levels in undifferentiated pSSCs. This indicates a heterogeneous population which is consistent with other adult stem cell populations [60], [72], [74].

One important finding of the study was that undifferentiated pSSCs express Sox2 and Oct3/4. This is interesting since the pluripotency of embryonic stem cells is governed via a regulatory complex of these two essential transcription factors. Sox2 and Oct3/4 enhance the expression of target genes like Nanog and Kfl4 in embryonic stem cells and therewith maintain their undifferentiated state [32], [75]. Furthermore, Sox2 is known to maintain a neuronal progenitor identity in the vertebrate central nervous system [76].

Recently it was demonstrated that the transcription factor Oct3/4 is not exclusively present in embryonic stem cells. It has been shown to be expressed in fetal and adult stem and progenitor cells from different tissue origins, in humans and pigs [17], [30], [77], [78]. Hence, it is not surprising that Oct3/4 was also detectable in undifferentiated pSSCs.

Besides Klf4 and c-Myc, Sox2 and Oct3/4 also play critical roles in inducing pluripotency in adult somatic cells. These cells known as induced pluripotent stem cells (iPSCs) provide a promising source of patient specific cells for cell replacement therapies as well as in vitro models for a variety of genetic diseases [79]–[81].

Due to the autogenous expression of Sox2 and Oct3/4, pSSCs would allow for a much easier and more effective induction of pluripotency by the induction of just one transcription factor, Kfl-4 or c-Myc. This has already been demonstrated using mouse neural progenitors which already express Sox2 [82], [83].

The progenitor marker nestin, the side population stem cells associated gene Bcrp1/ABCG2, the polycomb group repressor Bmi1 and the transcription factor Stat3 are also found to be expressed and therewith strengthen the stem cell/progenitor character of the isolated pSSC populations.

Undifferentiated human mesenchymal bone marrow stem cells already express the neuronal lineage markers β-III-tubulin and GFAP, and the smooth muscle marker α-SMA to a low extent before any differentiation [84]. Similar results were also found regarding the pSSCs investigated in this study. Except Dyce *et al.* who utilized cells of fetal origin no other investigation quantifed the intrinsic expression of lineage markers [30]. Hence, it is unknown if other adult stem cells from skin already express lineage marker genes at a similar level. The expression of lineage markers prior to induction of differentiation was also detected at later passages (data not shown). The expression of lineage markers before the initiation of the neuronal differentiation underlines the spontaneous differentiation of pSSCs during expansion; maybe induced by the fetal calf serum and due to the primary neurogenic conditions (bFGF, EGF, and B27) of the stem cell medium (SCM). Recent studies affirmed that bFGF has both mitogentic affects and reversibly inhibits differentiation depending on the differentiation state of the treated cells [72]. In addition, it is known that adult stem cells of different origin tend to differentiate spontaneously in the presence of FCS [18], [43], [85], [86]. Hence, we conclude that pSSCs represent a heterogeneous population of undifferentiated stem cells and more restricted progenitor cells.

Neuronal precursor cells are known to express the class VI intermediate filament protein nestin [87]. During differentiation into neuronal cell types nestin typically is down regulated [30], [65], [88]. A large sub-population of pSSCs expressed nestin (69.34%) before the induced differentiation. Since we observed that the number of nestin expressing cells decreased on an average of ∼15% but did not cease totally as expected and the neuronal lineage markers β-III-tubulin and GFAP increased following induced differentiation, we conclude a co-expression of these proteins. This co-expression suggests that the neuronal differentiation at the time of data collection was not completed and that the cells do not differentiate synchronously. However, the decrease of nestin expressing cells and the simultaneously observed increase of beta-III-tubulin and GFAP positive cells, and their change in morphology indicates that subpopulations of the pSSCs differentiated along the neurogenic lineage. Since the number of alpha-SMA positive cells increased dramatically while differentiation in NDM and subpopulations showed a morphology similar to smooth muscle cells we presume a neuro-muscular differentiation of pSSCs. The fact that only a subpopulation of the cells displayed this morphology but a high number of the differentiated cells expressed α-SMA further indicates a neuronal differentiation that is still in progress, since nestin and α-SMA are co-expressed during the development of neuronal cell types [89]. Other investigations have shown that nestin and GFAP are co-expressed during neuronal differentiation [65] and β-III-tubulin is co-expressed with GFAP and nestin in fetal astrocytes [90].

Finally, during a period of thirty days subpopulations of pSSCs differentiated into cell types of neuronal and muscular lineages while others still remained in a progenitor state, as indicated by the high expression levels of the respective proteins which suggests a co-expression (Fig. 4).

Additionally, pSSCs differentiated along the adipogenic lineage. This is demonstrated by two characteristic features of adipocytes, the formation of lipid droplets in the cytoplasm and the expression of leptin, a proteohormone that is mainly released by adipocytes and plays a key role in the energy expenditure of mammals [91].

Since pSSCs could be induced to differentiate to adipocyte-like cells when cultured under conditions for adipogenic differentiation of bone marrow derived MSCs, it might be possible to increase the amount of mature neuronal cell types by inducing neuronal differentiation via cytokines such as nerve growth factor or transforming growth factor beta [88], [92].

In summary, our investigation clearly demonstrates that adult porcine skin harbours a population of stem cell-like cells (pSSCs) that show characteristic features of mesenchymal stem cells. These pSSCs can be easily obtained by trypsin/EDTA digestion of porcine skin biopsies and are able to differentiate at least into cell types of mesodermal (smooth muscle cells, adipocytes) and ectodermal (neurons, astrocytes) origin. Further investigations are necessary to develop a method for the purification of the undifferentiated subpopulation of pSSCs and to maintain their undifferentiated state in a serum free medium. The high expansive potential of pSSCs *in vitro* and their intrinsic expression of Sox2 and Oct3/4 suggest pSSCs as interesting candidate cells for reprogramming into iPSCs.

## Materials and Methods

### Cell Isolation and Culture

Pig skin samples were obtained from a slaughterhouse (Elmars Metzgerei, GmbH & Co.KG, 54306 Kordel, Germany) after routine slaughter. Animals were killed in accordance to the guideline 93/119/EC of the European Community on the protection of animals at the time of slaughter or killing, dated December 22^nd^, 1993.

Porcine skin-derived stem cell-like cells (pSSCs) were isolated from porcine skin samples using a modified method by Toma *et al.* [16], previously described. Biopsies from the lower leg of half year old sows were washed two times with warmed HBSS (37°C, Gibco, Karlsruhe, Germany). The skin biopsies were then carefully dissected free of other tissue, cut into 2–3 mm^2^ pieces, washed again three times in HBSS (37°C) and digested overnight at 4°C in 0.25% trypsin/EDTA in PBS (Invitrogen, Karlsruhe, Germany). Subsequently skin pieces were incubated in 0.25% trypsin/EDTA for 30 min at 37°C. Trypsin activity was stopped with HBSS supplemented with 10% (v/v) FCS (PAA Laboratories, Coelbe, Germany). Skin pieces were then mechanically dissociated by vortexing and pipetting. The undigested skin remnants were discarded and the remaining cell suspension filtered through a 40 µm cell strainer (BD Falcon, Heidelberg, Germany). The cell suspensions were centrifuged at 160×g for five minutes and the resulting pellets resuspended in 10 ml HBSS for washing and again centrifuged at 160×g for five minutes. This process was repeated twice. Subsequently the cells were washed once with medium (DMEM/Ham's F12; 1∶1 both Biochrom, Berlin, Germany, 1 µg/ml fungizone, Invitrogen, Karlsruhe, Germany and 1% penicillin and 100 u streptomycin, PAA Laboratories, Coelbe, Germany). Each resulting cell pellet was resuspended in 1 ml stem cell medium (SCM): DMEM/Ham's F12 (1∶1) containing 10% FCS, 2% B27 (both PAA Laboratories, Coelbe, Germany), 40 ng/ml bFGF (Invitrogen, Karlsruhe, Germany), 20 ng/ml EGF (Sigma-Aldrich, Taufkirchen, Germany), 2 mM L-glutamine and antibiotics (PAA Laboratories, Coelbe, Germany).

Cells were counted with a hemocytometer and viability was verified by trypan-blue (Sigma-Aldrich, Taufkirchen, Germany) exclusion. Cells were seeded in 5 ml SCM with 2×10^5^ cells/cm^2^ growth surface and cultured in a 37°C, 5% CO_2_ tissue culture incubator (Heraeus, Langenselbod, Germany). The first medium-change was done following 1–4 days of culture, depending on donor variations regarding the cell attachment. Therewith the non-adhering cells were removed.

### Growth Rate and CFU Efficiency

To evaluate the growth rate of pSSCs, cells were seeded with 3,000 cells/cm^2^ in 25 cm^2^ tissue culture flasks (Sarsted, Nümbrecht, Germany) in SCM. Cells were detached using 0.25% trypsin/EDTA (Invitrogen, Karlsruhe, Germany) every seventh day and the cell number was determined. This procedure was repeated over a period of 126 days. After 126 days the experiment was stopped whereas pSSCs were still proliferating. The calculated population doublings were summarized to compute the cumulative population doubling (CPD) for each of the three cell strains.

To ascertain the CFU efficiency, cells were seeded at 10 cells/cm in culture dishes (Ø 6 cm) according to DiGirolamo *et al.* [70]. After 11 days, the cells were fixed in 95% isopropanol for 5 min and stained with 0.4% trypan-blue solution (Sigma-Aldrich, Taufkirchen, Germany) for 30 min. Thereafter cells were washed three times with 5 ml dH_2_O, the colonies were photographed (Canon IXUS 70) and subsequently measured using Image J analysis software (NIH; http://rsbweb.nih.gov/ij/). Only colonies with a diameter exceeding 1 mm were taken into account. CFU efficiency was expressed as the percentage of cells being capable of forming colonies.

### RNA Isolation and RT-PCR

Total RNA was isolated using trizol (Invitrogen, Karlsruhe, Germany) following the manufacturer's protocol. Any residual genomic DNA was eliminated by treatment with DNase I (Quiagen, Hilden, Germany). RNA concentration was photometrically assessed by measuring the absorbance at 260 nm. For reverse transcription, 2 µg RNA was used in a final volume of 20 µl containing 2.5 µM random hexameres, 25 U/µl MuLV reverse transcriptase (RT), RT-buffer (1X), 2 mM of dNTPs, 5 mM MgCl_2_, and 1 U/µl RNase-Inhibitor (all Applied Biosystems, Darmstadt, Germany).

Reverse transcription was carried out at 22°C for 10 min, 42°C for 15 min, 99°C for 5 min, 5°C for 5 min, and stored at 4°C until PCR analysis.

PCR amplification was completed using a Thermocycler (Biometra, Goettingen, Germany). The PCR consisted of 5 µl cDNA and 1 µl of each forward and reverse primer with a total volume of 50 µl.

The HPRT gene was amplified as an internal control for each PCR. The primers used for HPRT, Nestin, Sox2, Bcrp1, Bmi1, Stat3, Oct3/4 and Leptin have previously been published [7], [30].

The PCR was carried out with an initial denaturation for 3 min at 94°C followed by 35 cycles of 30 sec at 94°C, 30 sec corresponding annealing temperature, and 45 sec at 72°C. The final step consisted of three min of extension at 72°C. As a control for possible genomic DNA interference reactions were performed in which reverse transcriptase was omitted in the RT-step. PCR Products were run on 2% agarose gels (Serva, Heidelberg, Germany) and visualized with ethidium bromide (Roth, Karlsruhe, Germany) or SYBR Green (Quiagen, Hilden, Germany).

### Neurogenic Differentiation

pSSCs were grown in SCM until cultures were confluent. Subsequently, cells were rinsed with HBSS and cultured in neuronal differentiation medium (NDM) up to four weeks. NDM according to Dyce *et al.* [30] consists of DMEM/Ham's F12 (1∶1, both Biochrom, Berlin, Germany), 2 mM L-glutamine, 2% B27 (both PAA Laboratories, Coelbe, Germany), and antibiotics. The medium was changed every third day.

### Adipogenic Differentiation

Adipogenic differentiation was induced by culturing 90% confluent cultures in DMEM (Biochrom, Berlin, Germany) supplemented with 10% FCS (PAA Laboratories, Coelbe, Germany), 0.5 mM isobutyl-methylxanthine (IBMX) 10 µg/ml insulin, 1 µM dexamethasone, 100 µM indomethacin (all Sigma-Aldrich, Taufkirchen, Germany) and antibiotics [45]. The medium was changed every third day.

### Immunocytochemistry

pSSCs were plated on poly-D-lysin coated chamberslides (Nunc, Wiesbaden, Germany). After cell attachment or cell differentiation the cells were washed three times with HBSS and fixed and permeabilised for 5 min with methanol∶acteon (7∶3) at −20°C according to Kruse *et al.* [18]. After a subsequent washing step, unspecific domains were blocked for 15 min at room temperature with HBSS supplemented with 10% goat serum (PAA Laboratories, Coelbe, Germany). The cells were incubated with primary antibodies specific for nestin (mouse monoclonal, 1∶100; Invitrogen, Karlsruhe, Germany), beta-III-tubulin (mouse monoclonal, 1∶100), fibronectin (rabbit monoclonal, 1∶100), alpha smooth muscle actin (αSMA) (mouse monoclonal, 1∶100), glial fibrillary acidic protein (GFAP) (mouse monoclonal, 1∶100, all Sigma-Aldrich, Taufkirchen, Germany), medium neurofilament (NF-M) (monoclonal, 1∶100, Santa Cruz, Heidelberg, Germany) each diluted in HBSS containing 2% FCS. Incubation was processed over night in a humid chamber at 4°C.

After additional three times of washing with HBSS containing 2% FCS, primary antibody binding was detected by corresponding secondary antibodies goat anti-mouse IgG FITC conjugated (1∶100, Santa Cruz, Heidelberg, Germany) or rabbit anti-goat IgG R-PE conjugated (1∶100, Invitrogen, Karlsruhe, Germany). Incubation of the secondary antibody was processed at 37°C for 45 min followed by additional three washes with HBSS supplemented with 2% FCS. Stained cells were mounted with Vecatshield mouting medium (Linaris, Wertheim, Germany) containing DAPI to stain nuclei.

### Flow Cytometry

For quantification of cells expressing a given marker, flow cytometry analysis was performed. Cells were detached with 0.25% trypsin/EDTA, washed in PBS. Surface protein detection was realized by incubating the cells with antibodies against CD9, CD29, CD44, CD105 (all Abcam, Cambridge, UK), and CD90 (Lifespan Biosystems, supplied by Biozol, Muenchen, Germany) in the recommended dilutions of the manufacturers at 4°C for 30 min. To quantify intracellular protein expression, the cells were permeabilised and fixed using the BD Cytofix/Cytoperm™ Kit (BD Biosciences, Heidelberg, Germany). The fixed cells were incubated with primary antibodies specific for nestin (mouse monoclonal, 1∶100; Invitrogen, Karlsruhe, Germany), beta-III-tubulin (mouse monoclonal, 1∶100), and GFAP (mouse monoclonal, 1∶100, both Sigma-Aldrich, Taufkirchen, Germany) at 4°C for 30 min. After three additional washing steps with Perm/Wash (diluted 1∶10 in sterile H_2_O, BD Biosciences, Heidelberg, Germany), the cells were incubated with the secondary antibody (goat anit-mouse IgG FITC, 1∶100, Santa Cruz, Heidelberg, Germany) for 30 min at 4°C. For subtraction of background signals the cells were incubated with the corresponding isotype control. Analysis was performed using a flow cytometer (FACScalibur, BD Biosciences, Heidelberg, Germany). Ten thousand events per sample were acquired. Fluorescence gates were set on 2% and events corresponding to a fluorescence signal exceeding this percentage were considered as positive events. Results were analyzed and protein expression was quantified using CellQuest software (BD Biosciences, Heidelberg, Germany).

### Oil Red O Staining

Cells were washed with PBS, fixed in 10% formalin for one hour, washed in dH_2_O, and incubated with filtered 0.3% oil red O (Sigma-Aldrich, Taufkirchen, Germany) diluted in 60% isopropanol for 10 min. The oil red O stain was aspirated and the dishes were washed with H_2_O until the solution was clear. Bright field microscopic pictures were taken immediately after staining.
